# A paradigm shift in liquid cooling by multitextured surface design

**DOI:** 10.1016/j.xinn.2022.100222

**Published:** 2022-03-04

**Authors:** Ying Zhou, Pingan Zhu

**Affiliations:** 1 Department of Mechanical Engineering, City University of Hong Kong, Hong Kong 999077, China

**Figure 1:**
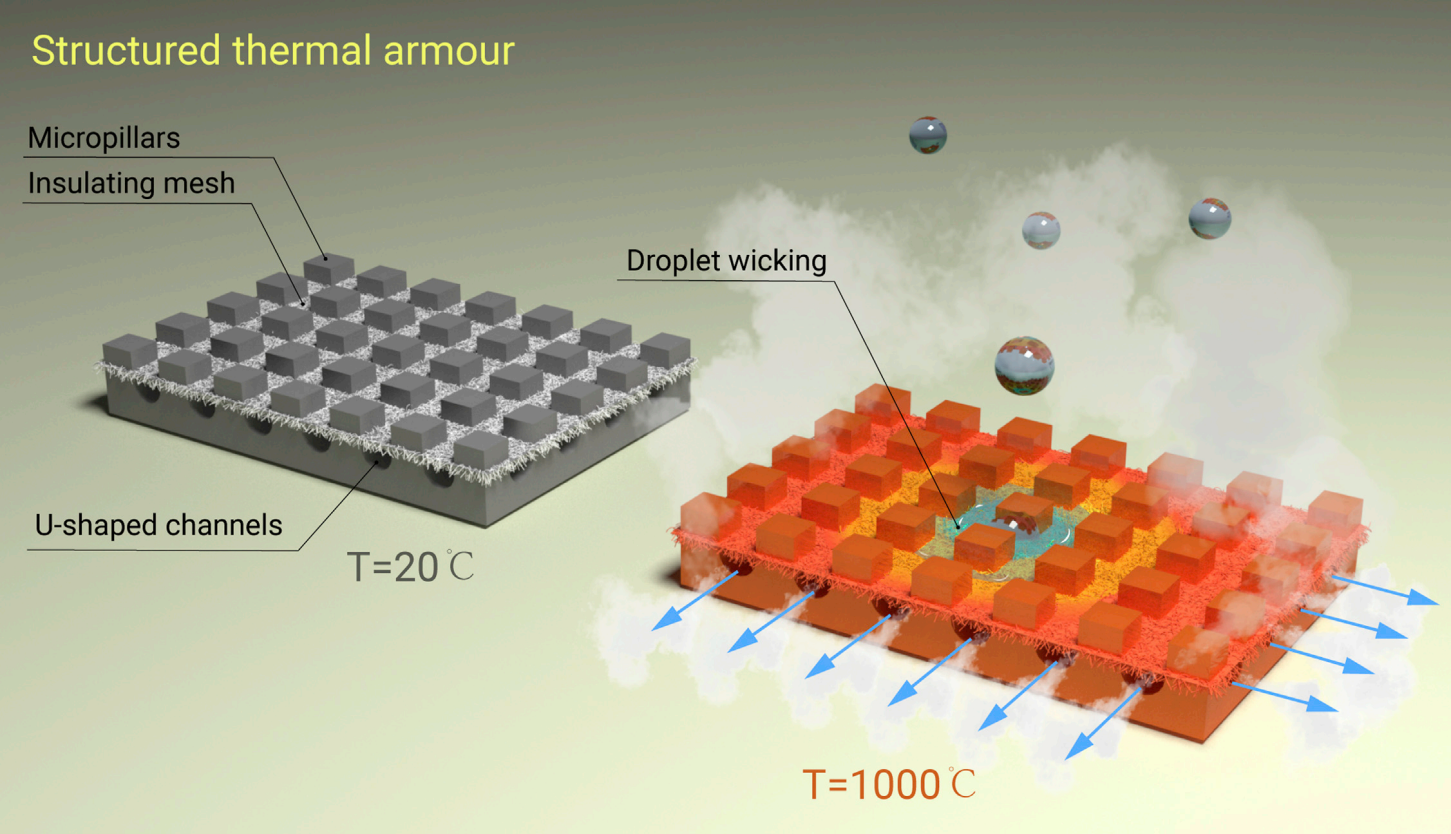
Structured thermal armour (STA) inhibiting the Leidenfrost effect The schematic of the STA design (left, *T* = 20°C), consisting of an array of steel micropillars with an insulating mesh (silica porous membrane) and U-shaped channels. Upon cooling by water spraying (right, *T* = 1000°C), droplets are absorbed by the insulating mesh and evaporate on steel micropillars, and U-shaped channels evacuate the produced vapours.

Effective thermal management of high-temperature systems, including rapid cooling and accurate temperature control, has been a necessity in many industrial applications, such as electronics, spacecraft, and nuclear power plants. The well-established water spraying and soaking are among the most commonly used strategies for thermal cooling. Direct contact of water with a hot surface releases the latent energy by the liquid-vapour phase change, presenting a remarkable cooling strategy. However, when the surface temperature exceeds a certain threshold, a stable vapour film is sandwiched between the solid and the liquid due to rapid water evaporation, by which droplets hover over the surface rather than make physical contact with it. This levitation of droplets on hot solids, named after Johann Gottlob Leidenfrost, is known as the Leidenfrost effect.^1^ Recently, Jiang et al.^2^ reported a multitextured design of structured thermal armour (STA; Figure 1), capable of inhibiting this effect at temperatures up to 1,150°C.

The Leidenfrost point (LFP) represents the temperature at which the heat flux is a minimum, above which the Leidenfrost effect is triggered. With the Leidenfrost effect, film boiling occurs where heat transfer from the hot surface to the liquid is by conduction and radiation through the vapour. In this regime, the hot surface is completely covered by an insulating vapour blanket, resulting in a lower heat flux and a long time of water evaporation. The occurrence of the Leidenfrost effect overheats the surface uncontrollably, leading to a boiling crisis. As such, many efforts have been devoted to inhibiting the Leidenfrost phenomenon for efficient heat transfer during high-temperature cooling.

A straightforward strategy to suppress the Leidenfrost state is to increase the LFP. There are two typical ways for this purpose. One way is to change the physicochemical properties of the liquid, for example, by using a high-boiling-point liquid and varying the density of the liquid. Another way focuses on modifying the surface parameters such as the roughness, wettability, and porosity. Compared with varying the liquid properties, engineering the surface properties is more appealing and relevant in practice.

Given that the Leidenfrost effect arises mainly from the formation of a stable vapour film, one strategy is to break down this film and bring the liquid back into contact with the hot surface for restoring the rapid evaporation.^3^ Stable vapour films tend to form on smooth surfaces. Nevertheless, the film is prone to be disturbed by structures on rough surfaces where the solid-liquid contact becomes rugged. Previous studies have demonstrated the disruption of vapour films by micro/nanotextured surfaces, which can shift the LFP by up to 175°C relative to the polished surface.^3^

Micro/nanotextured surfaces fail in inhibiting the Leidenfrost effect when the surface temperature goes even higher. The state of a cooling droplet is governed by a competition between the capillary pressure, which promotes droplet wetting, and the vapour pressure, which pushes up the droplet for dewetting. An increase in temperature augments the dewetting vapour pressure, thus lifting the droplet to trigger the Leidenfrost phenomenon. The vapour pressure can be reduced by lowering the resistance of the vapour flow. Guided by this rule, Farokhnia et al.^4^ designed a decoupled hierarchical structure composed of a nanomembrane assembled on top of a deep micropillar array, by which the LFP can be raised to 570°C. The mismatch in structure geometry decouples droplet wicking and vapour evacuation where the capillary pressure is governed by nanopores and the vapour pressure is by micropillars. A remarkable increase of LFP to 700°C was achieved by Vakarelsk et al.^5^ using superhydrophilic surfaces coated with a 1-μm-thick silicon oxide structure layer. By quenching a high-temperature superhydrophilic sphere in water, they observed nucleate boiling with the collapse of the vapour layer and release of bubbles from the solid surface.

It was a long-standing and grand challenge to achieve an LFP exceeding 1,000°C before the work by Jiang et al., even though the temperature of 1,000°C is highly relevant in many practical scenarios. For example, the temperature of metal smelting and quenching can be as high as 1,000°C, and the temperature of fuel rods in the nuclear reactor core is well above 1,000°C. To inhibit the Leidenfrost effect above 1,000°C for sustained thermal cooling, the multitextured STA is designed with three features (Figure 1): the thermally conductive steel micropillars acting as thermal bridges, the thermally insulating silica porous membrane serving as a wicking mat for cooling droplets, and the U-shaped channels providing passages for unimpeded vapour evacuation.^2^ The capillary wicking effect is effectively brought into play by the superhydrophilicity and low thermal conductivity of the silica membrane. The wicking greatly disrupts the formation of the vapour film, allowing the highly conductive steel pillars to be in direct contact with the droplet for rapid evaporation and thus preserving the extremely high heat dissipation. More subtly, the U-shaped channels efficiently direct the vapour to flow out of the surface with reduced resistance, allowing subsequent drops to remain in constant contact with the hot surface, instead of being disturbed by the vapour.

The most striking feature of STA is the use of multitextured materials with contrasting properties. The porous membrane has a low thermal conductivity in STA, which, at a first glance, seems to be counterintuitive because efficient heat transfer is normally associated with thermally conductive materials. Indeed, the key to inhibiting the Leidenfrost effect at high temperatures is attributed to the contrast in thermal conductivity between the membrane and the steel. Owing to its low thermal conductivity, the silica membrane has a lower temperature than steel pillars so droplet wicking is restricted on the membrane while vapour generation mainly occurs on steel pillars, by which the two processes are disentangled. The STA is advantageous in several other aspects. For instance, this structure enables accurate control over the surface temperature, since the cooling effect can be regulated by changing the water flux, the membrane pore size, and the channel depth. Moreover, the porous membrane is nested inside the rigid, thick steel pillars, which can physically protect the membrane, rendering the design mechanically robust. In addition, the surface can be made flexible and conformable to complex-shaped surfaces, substantially expanding the application horizons.

The work by Jiang et al. will inspire many future explorations on liquid cooling. First, it would be desired to have an ultra-high heat flux (e.g., >10^7^ W m^−2^) exceeding the typical value of ∼10^6^ W m^−2^ at high temperatures for highly efficient heat transfer in demanding applications, including spacecraft and nuclear power generation. Second, it is of great significance to explore the system's performance in diverse environments, such as under extreme pressure or microgravity to facilitate applications in outer space. More efforts can be devoted to extending the STA to a wider choice of materials for improved thermal performance and multifunctionality. Last but not least, broader applications are envisioned by upgrading the STA to minimize its influence on the appearance of coated surfaces.

The increasing demand for energy consumption by human society constantly poses new challenges to thermal management. Suppressing the Leidenfrost effect for efficient thermal cooling will have a profound impact on high-temperature applications. Jiang and colleagues' work could provide a promising solution to the boiling crisis. The excellent achievement results from the synergy of multitextured functional units for decoupling the three processes: a silica membrane for droplet wicking, steel pillars for vapour generation, and U-shaped channels for vapour evacuation. The design strategy could also inspire new solutions to problems in various fields not limited to heat transfer, thus opening up new opportunities for future applications.
